# MeCP2 Dosage Control in Rett Syndrome: Non-Coding RNA-Based and Epigenetic Strategies for Safer Gene Therapy

**DOI:** 10.3390/ncrna12040025

**Published:** 2026-07-22

**Authors:** Ilyas M. Kabdesh, Albert A. Rizvanov, Yana O. Mukhamedshina

**Affiliations:** 1 OpenLab «Gene and Cell Technologies», Institute of Fundamental Medicine and Biology, Kazan (Volga Region) Federal University, 420021 Kazan, Russia; albert.rizvanov@kpfu.ru (A.A.R.); yomuhamedshina@kpfu.ru (Y.O.M.); 2 Division of Medical and Biological Sciences, Tatarstan Academy of Sciences, 420013 Kazan, Russia; 3 Department of Histology, Cytology and Embryology, Kazan State Medical University, 420008 Kazan, Russia

**Keywords:** Rett syndrome, MeCP2, gene therapy, AAV, non-coding RNA, miRNA, X-chromosome reactivation, epigenome editing, CRISPR/dCas9

## Abstract

Rett syndrome (RTT) is a severe X-linked neurodevelopmental disorder that is caused in most cases by pathogenic variants in *MECP2*, the gene encoding methyl-CpG-binding protein 2 (MeCP2). Despite substantial progress in the development of gene therapy, restoring *MECP2* expression remains challenging because MeCP2 is highly dosage-sensitive. Both deficiency and excessive expression of this protein are associated with severe neurological abnormalities. This makes simple viral vector-mediated replacement of *MECP2* potentially unsafe and underscores the need for multilayered systems that control transgene expression. This review discusses current and emerging strategies for regulating MeCP2 expression in RTT, with an emphasis on non-coding RNA-based and epigenetic mechanisms. Particular attention is given to the limitations of conventional AAV-mediated gene therapy, the use of cell-specific and endogenous promoters, miRNA-regulated elements, autoregulatory systems, and post-transcriptional control of *MECP2* expression. Strategies for reactivating the inactive X chromosome are also discussed, including XIST-dependent regulation and epigenome editing. In addition, the review considers CRISPR-mediated regulation, selective epigenetic activation, and combined therapeutic platforms that integrate viral delivery, RNA-dependent post-transcriptional control, and endogenous gene regulation. Overall, clinically applicable gene therapy for RTT will likely need to move beyond simple *MECP2* replacement and instead rely on precise cell- and dose-dependent regulation of its expression. Non-coding RNA and epigenetic mechanisms represent important layers of such control and may contribute to the development of safer gene therapy strategies for RTT.

## 1. Introduction

**Figure 1:**
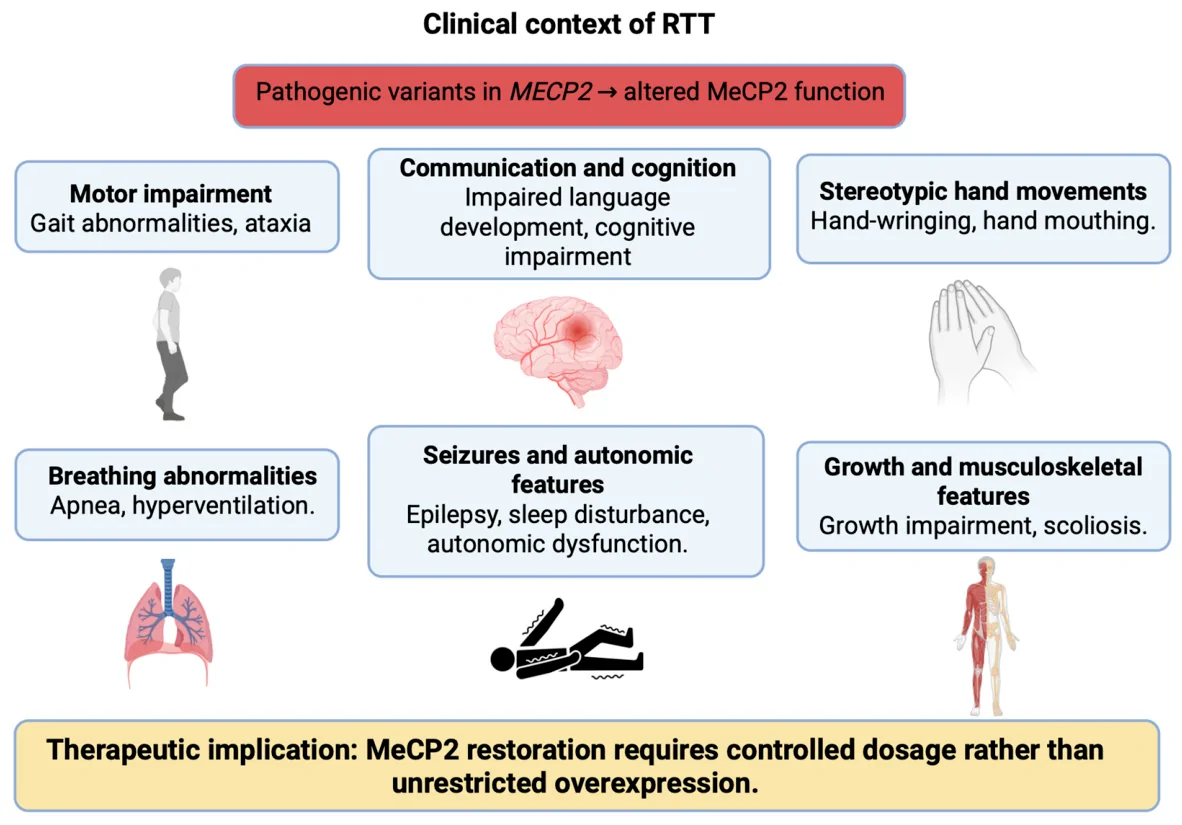
Clinical context of RTT. Schematic representation of the main clinical patterns associated with RTT and their relationship to impaired MeCP2 function. RTT is typically characterized by regression of acquired skills, loss of purposeful hand use, hand stereotypies, gait abnormalities, breathing disturbances, seizures, and variable cognitive and autonomic manifestations. The figure is intended to provide a general clinical context and does not imply that all manifestations are present in every patient.

Rett syndrome (RTT) is a neurodevelopmental disorder clinically characterized by regression of previously acquired skills, including purposeful hand use and spoken language, gait abnormalities, and the emergence of stereotypic hand movements. These features are included in the revised diagnostic criteria for classic RTT [1]. Pathogenic variants in the X-linked gene *MECP2*, which encodes methyl-CpG-binding protein 2 (MeCP2), were identified in patients with RTT, linking the disorder to impaired MeCP2-dependent regulation of chromatin and transcription [2,3,4,5]. Analysis of the Rett Networked Database further reported significant differences in clinical severity among mutation groups, supporting genotype-phenotype correlations between *MECP2* pathogenic variants and clinical phenotype [6]. MeCP2 was characterized as a protein capable of binding methylated CpG dinucleotides and participating in transcriptional repression through interactions with histone deacetylases and the SIN3A corepressor complex [7]. Recent studies further suggest that MeCP2 deficiency may affect broader cellular homeostasis, including DNA damage responses, genomic maintenance, cellular stress, and metabolic pathways, indicating that RTT pathogenesis cannot be reduced to simple transcriptional misregulation alone [8,9,10]. In this review, *MECP2* is used when referring to the human gene or gene therapy strategies in a general translational context, whereas *Mecp2* is used for mouse genes and mouse models. MeCP2 refers to the protein. The main clinical features of RTT are schematically summarized in Figure 1.

After *MECP2* was identified as the major gene associated with RTT, restoration of MeCP2 function began to be considered a logical therapeutic strategy. This concept was supported by studies in which restoration of *Mecp2* expression in mice was associated with attenuation of several RTT-like neurological manifestations [11]. However, these early data were obtained in an experimental model and do not imply that simple restoration of MeCP2 expression in patients would be sufficient or safe. Subsequent experimental studies in transgenic animals showed that, in RTT, it is essential not only to restore protein function but also to control the level of MeCP2 expression in cells of the nervous system [12].

This feature distinguishes RTT from many disorders in which the therapeutic rationale may be reduced to delivery of a functional copy of a defective gene. In RTT, this strategy is complicated by several factors: MeCP2 is a dosage-sensitive protein, the disease in female patients develops in the context of X-linked cellular mosaicism, and vector-mediated transgene delivery can result in uneven expression levels across cells [13]. Therefore, gene therapy for RTT requires not only efficient delivery of *MECP2*/*Mecp2*, but also mechanisms that limit the risk of both insufficient and excessive MeCP2 expression.

In this context, regulatory layers that act after delivery of the coding sequence or target the endogenous locus are of particular interest. Non-coding RNA-based mechanisms can contribute to post-transcriptional restriction of transgenic mRNA expression [14,15], whereas the long non-coding RNA XIST (X-inactive specific transcript) is a key component of the epigenetic program of X-chromosome inactivation and is therefore relevant to strategies aimed at reactivating endogenous *MECP2*/*Mecp2* from the inactive X chromosome [16,17]. Although these mechanisms do not eliminate all limitations of gene therapy, they provide a framework for moving from simple transgene expression toward more controlled strategies for regulating MeCP2.

The aim of this review is to critically examine strategies for controlling MeCP2 dosage in RTT, with an emphasis on non-coding RNA-based and epigenetic approaches. Particular attention is given to regulatory layers that may reduce the risk of MeCP2 expression falling outside a therapeutically acceptable range, limitations already identified in preclinical studies, approaches that have advanced to clinical evaluation, and safety issues that remain central to the further translation of these strategies.

## 2. The Problem of MeCP2 Dosage Sensitivity

One of the central features of RTT is the dependence of the phenotype on the level of functional MeCP2. Mice with disrupted Mecp2 develop progressive neurological abnormalities, including motor deficits, tremor, breathing disturbances, and reduced survival, making these models useful tools for studying the consequences of MeCP2 deficiency in vivo [11]. Subsequent Cre-LoxP-dependent reactivation of Mecp2 in a mouse model carrying a lox-Stop cassette in the endogenous Mecp2 locus was associated with attenuation of several neurological manifestations, including abnormalities already established in adult mice [11]. Thus, under experimental conditions, some phenotypic manifestations associated with MeCP2 deficiency could be modified after restoration of Mecp2 expression; however, these data do not allow direct assessment of the extent to which RTT is reversible in patients.

The sensitivity of MeCP2 to gene dosage is further supported by evidence on the consequences of increased MeCP2 expression. In male patients, duplication of the Xq28 region containing *MECP2* has been associated with severe cognitive impairment, hypotonia, seizures, and progressive neurological manifestations [18]. This condition is not RTT itself, but it is important for evaluating the safety of replacement approaches, because it shows that increased dosage of a genomic region containing *MECP2* can be associated with an independent pathological phenotype.

Experimental models also indicate that uncontrolled MeCP2 overexpression may be unsafe. In transgenic mice expressing human MeCP2 from a large genomic clone, increased MeCP2 levels were associated with a progressive neurological deficit. Some animals developed seizures, hypoactivity, and premature death, leading the authors to conclude that MeCP2 levels must be tightly regulated in vivo [12]. In another in vivo mouse model, Tau-*Mecp2*, characterized by neuron-restricted MeCP2 overexpression, animals exhibited impaired motor coordination, increased anxiety-like behavior, and deficits in learning and memory. These behavioral abnormalities were accompanied by impairments in long-term potentiation and short-term synaptic plasticity. In addition, cultured hippocampal neurons derived from Tau-*Mecp2* mice showed an increased frequency of miniature excitatory postsynaptic currents (EPSCs) without changes in miniature inhibitory postsynaptic currents (IPSCs), suggesting a predominant effect of MeCP2 overexpression on excitatory synaptic transmission [19]. The available data do not define a universal safe threshold of MeCP2 expression in humans, but they support the view that excess MeCP2 should be considered a potential risk factor in the development of replacement therapies [20,21,22].

A recent study further refines this concept by indicating that the biological consequences of ectopic MeCP2 expression may depend on developmental timing and cellular context. Luoni et al. (2026) showed that MeCP2 overexpression induced pronounced transcriptional alterations in neural progenitor cells, whereas mature neurons showed a more limited response to comparable ectopic MeCP2 expression in both mouse and human experimental systems [23]. These findings suggest that the consequences of MeCP2 overexpression are not uniform across all cellular states and that the timing of transgene expression may influence therapeutic risk. Importantly, however, this does not eliminate the need for regulated expression systems, because long-term exposure, non-neuronal cell types, peripheral expression, and the mosaic cellular context of RTT patients remain important safety considerations.

Taken together, these observations indicate that simply achieving MeCP2 expression is not sufficient for RTT therapy. A therapeutic construct must account for the need to control protein levels in a cell- and context-dependent manner, because both MeCP2 deficiency and increased MeCP2 expression have been associated with pathological phenotypes in clinical and experimental observations. In this context, the therapeutic goal is not to maximize expression, but to achieve a level of MeCP2 that is compatible with functional correction while minimizing the risk of overexpression.

An additional complication arises from the X-linked nature of RTT in female patients. Due to random X-chromosome inactivation, different cells may express either the mutant or the functional copy of *MECP2*, resulting in cellular mosaicism in the level of functional MeCP2 [24,25,26]. Therefore, additional transgene expression may have different consequences across cell populations: in cells deficient in functional MeCP2, it may be therapeutically beneficial, whereas in cells already expressing functional MeCP2, it may increase the risk of excessive expression [16,27].

From a practical standpoint, MeCP2 dosage sensitivity imposes several requirements on therapeutic strategies. Transgene expression must be restricted not only by cell type, but also by expression level within target cells. Variability in transduction must also be considered, because different cells may receive different numbers of vector copies. These considerations explain the shift from simple replacement constructs toward multilayered control systems that incorporate promoter regulation, post-transcriptional RNA-mediated elements, and strategies aimed at restoring expression from the endogenous *MECP2* locus.

## 3. AAV-Mediated Delivery and Promoter Control

AAV-mediated delivery of *MECP2* has been investigated as a replacement-based strategy for RTT because it enables delivery of a coding sequence to cells of the nervous system and can support long-term transgene expression. However, the dosage sensitivity of MeCP2 limits the applicability of a standard approach [28] based solely on delivering a functional gene copy and driving its expression without additional regulatory mechanisms. Preclinical studies of AAV9-mediated *MECP2*/*Mecp2* delivery have shown that gene transfer can attenuate selected phenotypic manifestations in mice with MeCP2 deficiency. In the study by Gadalla et al., neonatal intracranial delivery of ssAAV9/CBA-MECP2 to male *Mecp2*-null mice increased median survival from 9.3 to 16.6 weeks and reduced the severity of RTT-like phenotypes compared with the AAV9/GFP control group. At the same time, a separate systemic delivery experiment in WT mice demonstrated an important limitation of intravenous administration. After intravenous administration, the AAV9 vector accumulated predominantly in the liver and spleen, and AAV9/MECP2-treated mice showed increased alanine aminotransferase (ALT), which the authors interpreted as possible liver dysfunction associated with high vector load and MeCP2 expression in liver cells [29].

Subsequent studies further showed that the outcome of AAV-*MECP2*/*Mecp2* therapy depends on dose, route of administration, and vector design. Sinnett et al. evaluated intracisternal administration of a first-generation AAV9/hMECP2(v1) vector as a strategy to shift transgene expression toward the CNS. Although this approach increased median survival in *Mecp2*-/y mice, it was accompanied by adverse phenotypes in both mutant and WT mice, indicating the risk of MeCP2 overexpression when vector-mediated expression occurs in cells already containing endogenous protein. Dose reduction improved tolerability but reduced therapeutic benefit. The authors therefore modified the cassette by incorporating additional endogenous regulatory elements, including an extended promoter fragment and a modified 3′ UTR (untranslated region) with miRNA-binding sites. Intracisternal administration of AAV9/hMECP2(v2) at lower doses extended survival and did not increase serum markers of hepatotoxicity at 3–4 weeks after injection; however, it did not produce sustained correction of RTT-like behavioral phenotypes [30].

An attempt to approximate transgene expression to the endogenous pattern was also implemented using a minimal endogenous *Mecp2* promoter. Systemic administration of an AAV9 construct driven by this promoter was associated with a narrow therapeutic window: lower doses increased survival in mice lacking MeCP2 but did not produce robust correction of all RTT-like neurological signs, whereas higher doses were again associated with hepatotoxicity [31]. Similarly, in *Mecp2*-heterozygous mice, intravenous delivery of AAV9-*Mecp2* was associated with signs of liver injury at higher doses, whereas a lower dose produced only a limited functional effect, primarily a reduction in apnea frequency [32]. These findings indicate that promoter selection and dose adjustment can reduce some risks, but they do not fully prevent peripheral expression or guarantee separation between therapeutic and toxic effects.

Promoter control should therefore be viewed as a basic but limited layer of regulation in this context. The use of weaker, cell-oriented, or endogenous regulatory elements may reduce the baseline transcriptional activity of the cassette and limit off-target expression. However, a promoter is not a sensor of MeCP2 abundance in an individual cell. It does not compensate for variability in vector copy number, distinguish cells with insufficient MeCP2 from cells already expressing functional MeCP2, or provide full self-regulation after transduction. Therefore, promoter specificity is important for the rational design of *MECP2*/*Mecp2* replacement constructs, but it cannot be considered a stand-alone solution to the dosage problem.

Promoter control can also be used to restrict expression to defined cell populations. For example, hSyn/SYN1 and Hb9 promoters have been used to direct neuronal AAV-mediated expression, including after intrathecal delivery of AAV9 vectors to the CNS [33]. Activity-dependent regulatory elements should be considered separately. These include regulatory sequences of immediate early genes, such as *Fos*/*c-Fos*, *Arc*/*Arg3.1*, *Egr1*/*Zif268*, and *Npas4*, which are used to label activated neuronal populations [34,35]. Conceptually, such elements could link therapeutic transgene expression to the functional state of a neuron. However, published data demonstrating their use for regulated *MECP2*/*Mecp2* delivery in RTT are currently lacking. Thus, these systems should presently be regarded as a potentially attractive, but experimentally unvalidated, form of promoter-level regulation for RTT.

Overall, AAV-mediated *MECP2*/*Mecp2* delivery in preclinical models demonstrates functional potential but remains limited by a narrow therapeutic window, dose-associated toxicity, peripheral expression, heterogeneous transduction, and incomplete cell-level dosage control [36,37]. Promoter control can reduce some of these risks, but it cannot directly adjust MeCP2 abundance after transduction. These limitations have driven the development of multilayered regulatory systems, including post-transcriptional control mediated by miRNA-responsive elements. Because these elements act at the level of transgenic mRNA, they are particularly relevant to regulated MeCP2 replacement strategies and are discussed in detail in the following section.

## 4. Post-Transcriptional Control Using miRNA-Responsive Elements

Post-transcriptional control is currently one of the most relevant directions for developing regulated *MECP2* replacement strategies, because it uses RNA-dependent mechanisms to restrict transgene expression. In contrast to promoter control, which sets the baseline transcriptional activity of an expression cassette, miRNA-responsive elements act after mRNA has been generated [38,39]. Their principle is based on the incorporation of microRNA-binding sites into the transgenic transcript, most often within the 3′ UTR. After a microRNA is loaded into the RNA-induced silencing complex (RISC), this complex recognizes complementary mRNA sequences and can reduce protein production by repressing translation or accelerating mRNA degradation [40]. In gene therapy, this principle is used to limit transgene expression in cells or tissues in which the corresponding endogenous miRNAs are active; incorporation of miRNA target sites into viral vectors can reduce unwanted transgene expression in specific cell populations or tissues [39]. However, such a system cannot serve as a direct sensor of MeCP2 concentration, because its effectiveness depends on the selected miRNA-binding sites, the abundance of the corresponding miRNAs, RISC activity, cell type, age, tissue state, and transduction level.

The most direct example of this approach in an RTT model is miRARE, a microRNA-responsive autoregulatory element. miRARE was incorporated into a self-complementary AAV construct carrying miniMECP2, a truncated version of MeCP2 that leaves additional space in the AAV cassette for regulatory elements. In the study by Sinnett et al., miRARE was evaluated in adolescent mice aged 4–5 weeks using intracisternal and intrathecal administration. Initially, unregulated AAV9/miniMECP2 and PHP.B/miniMECP2 constructs caused signs of toxicity in WT mice, including aggravated hindlimb clasping and increased aggregate phenotype severity scores, indicating a risk of miniMECP2 overdosing in the presence of endogenous MeCP2. Subsequent comparison of unregulated AAV9/miniMECP2 and regulated AAV9/miniMECP2-miRARE after intrathecal administration showed that miRARE reduced miniMECP2-associated adverse effects in WT mice while preserving therapeutic benefit in knockout mice: AAV9/miniMECP2-miRARE increased median survival in knockout mice from 9.6 to 15 weeks at a dose of 1 × 10^12^ vg/mouse (vector genomes per mouse). Thus, in this study, miRARE did not act as a physiological sensor of MeCP2 abundance, but rather as an engineered miRNA-responsive expression-limiting element designed to reduce the risk of excessive miniMECP2 expression after AAV delivery [16]. This is particularly relevant in the RTT context, because the presence of cells retaining a functional *MECP2* copy is one of the reasons for the risk of overexpression in patients.

When describing the mechanism of miRARE, it is important to avoid excessive simplification. This element does not fully restore the natural regulation of *MECP2*/*Mecp2* and does not make transgene expression entirely physiological. It is more accurately viewed as an engineered post-transcriptional limiter that reduces the risk of excessive accumulation of transgenic mRNA or protein under specific conditions. Therefore, the main significance of miRARE lies in reducing the risk of overexpression and expanding the range of doses at which the construct maintains an acceptable safety profile in preclinical models. Accordingly, the effectiveness of this element should not be evaluated separately from the vector, but in the context of a specific expression cassette, dose, route of administration, and target transduced cell populations.

A translational extension of this strategy is TSHA-102, a human-ready regulated miniMECP2 gene therapy. In a published preclinical study, TSHA-102 was evaluated in male *Mecp2*-/y knockout mice after intracerebroventricular administration at P2 and intrathecal administration at P7, P14, and P28, including separate groups receiving immunosuppression. A safety assessment was also performed in adult female *Mecp2*+/− mice. After P2 intracerebroventricular administration, TSHA-102 increased median survival of knockout mice by more than 300% compared with the vehicle-treated knockout group and reduced aggregate RTT severity scores at several time points. When administered intrathecally at later postnatal stages, TSHA-102 improved body weight, increased survival, and affected selected respiratory parameters, including respiratory rate, expiratory time, inspiratory time, and apnea-related measures. However, its efficacy varied depending on age at administration, dose, and the use of immunosuppression. In addition, some comparisons were limited by group size, data variability, and the potential influence of immunosuppression used in selected experimental series. Biodistribution and expression analyses confirmed the presence of vector DNA and miniMECP2 mRNA in the CNS after TSHA-102 administration [41]. At the same time, precise quantification of miniMECP2 mRNA was limited because miRARE markedly reduces accumulation of the transgenic transcript, and some measurements may have been near the lower limit of quantification. Thus, low mRNA levels may reflect the activity of the regulatory element rather than absence of expression. For this reason, TSHA-102 should be regarded as an important preclinical example of regulated miniMECP2 therapy, but not as evidence that miRNA-responsive control fully solves the problem of MeCP2 dosage.

In addition to the published mouse-model data, TSHA-102 has advanced into clinical evaluation as a regulated miniMECP2/miRARE strategy. The REVEAL program is evaluating a single intrathecal administration of TSHA-102 in female patients with RTT, with the pivotal cohort focused on females aged 6 to <22 years, whereas the ASPIRE study evaluates girls aged 2 to <4 years [ClinicalTrials.gov: NCT05606614, NCT06152237, NCT07480564]. According to publicly reported updates from the sponsor, dosing has been completed in the REVEAL pivotal cohort, while the younger cohort remains under evaluation. Longer-term data reported from REVEAL Part A described functional gains across several core RTT domains, including communication, gross motor function, and fine motor or hand-related skills. These findings are clinically encouraging because they suggest multidomain effects rather than improvement limited to a single outcome measure. However, they should be interpreted in the context of an open-label rare-disease development program and in the absence of a full peer-reviewed clinical report with long-term follow-up. Importantly, clinical improvement does not directly demonstrate that MeCP2 expression is physiologically normalized in every relevant CNS cell population. Therefore, the main long-term questions for TSHA-102 are whether miniMECP2 provides durable functional benefit, whether miRARE-mediated restriction remains stable across developmental stages and CNS cell types, and whether delayed signs of insufficient expression or overexpression emerge during extended follow-up.

A separate line of RNA-mediated control is represented by synthetic dosage-compensating miRNA circuits based on an incoherent feed-forward loop (IFFL) architecture. In such systems, expression of the therapeutic gene is coupled to expression of a limiting miRNA component, so that increased cassette activity also increases the regulatory signal that suppresses excessive accumulation of the corresponding transcript [42]. For *Mecp2*, this principle was applied by Flynn et al. (2024), who used synthetic miRNA circuits to reduce the dependence of MeCP2 levels on delivery dose both in vitro and in vivo in mice [43]. Importantly, the integrated miRNA used in this approach is not a copy or direct analogue of an endogenous miRNA. The authors used an engineered miRNA expression cassette based on the miR-E backbone, which generates a miRNA complementary to an artificial target sequence derived from *Renilla* luciferase and designed to be orthogonal to the human genome. Thus, in this IFFL system, repression is mediated by a synthetic miRNA paired with its engineered target sites in the transgenic mRNA, rather than by an endogenous miRNA pathway.

This strategy differs mechanistically from miRARE. miRARE relies on endogenous miRNA/RISC activity acting on miRNA-responsive sites embedded in the miniMECP2 cassette, whereas an IFFL circuit introduces a synthetic buffering module that links transgene expression to a corresponding inhibitory miRNA component. Thus, miRARE functions primarily as an embedded post-transcriptional limiter, whereas IFFL is designed to buffer intercellular differences in expression levels. At present, this approach remains experimental, and its developmental status in relation to clinical translation remains unclear.

A related direction is represented by self-regulating gene therapy developed for full-length human *MECP2* and based on a miRNA-mediated regulatory circuit designed to address MeCP2 dosage sensitivity. Ross et al. (2025) described this system as Expression Attenuation via Construct Tuning (EXACT), a self-contained miRNA-based feed-forward loop incorporated into the therapeutic cassette [44]. In this design, a single promoter drives expression of a full-length human *MECP2* transgene together with a non-naturally occurring miRNA sequence, generated from an embedded miRNA scaffold. This regulatory miRNA acts through complementary recognition sites located in the 3′ UTR of the transgenic *MECP2* mRNA, thereby limiting excessive transgene expression. The authors also showed that the circuit could be tuned by modifying cassette elements such as the number and sequence of miRNA-binding sites.

**Figure 2:**
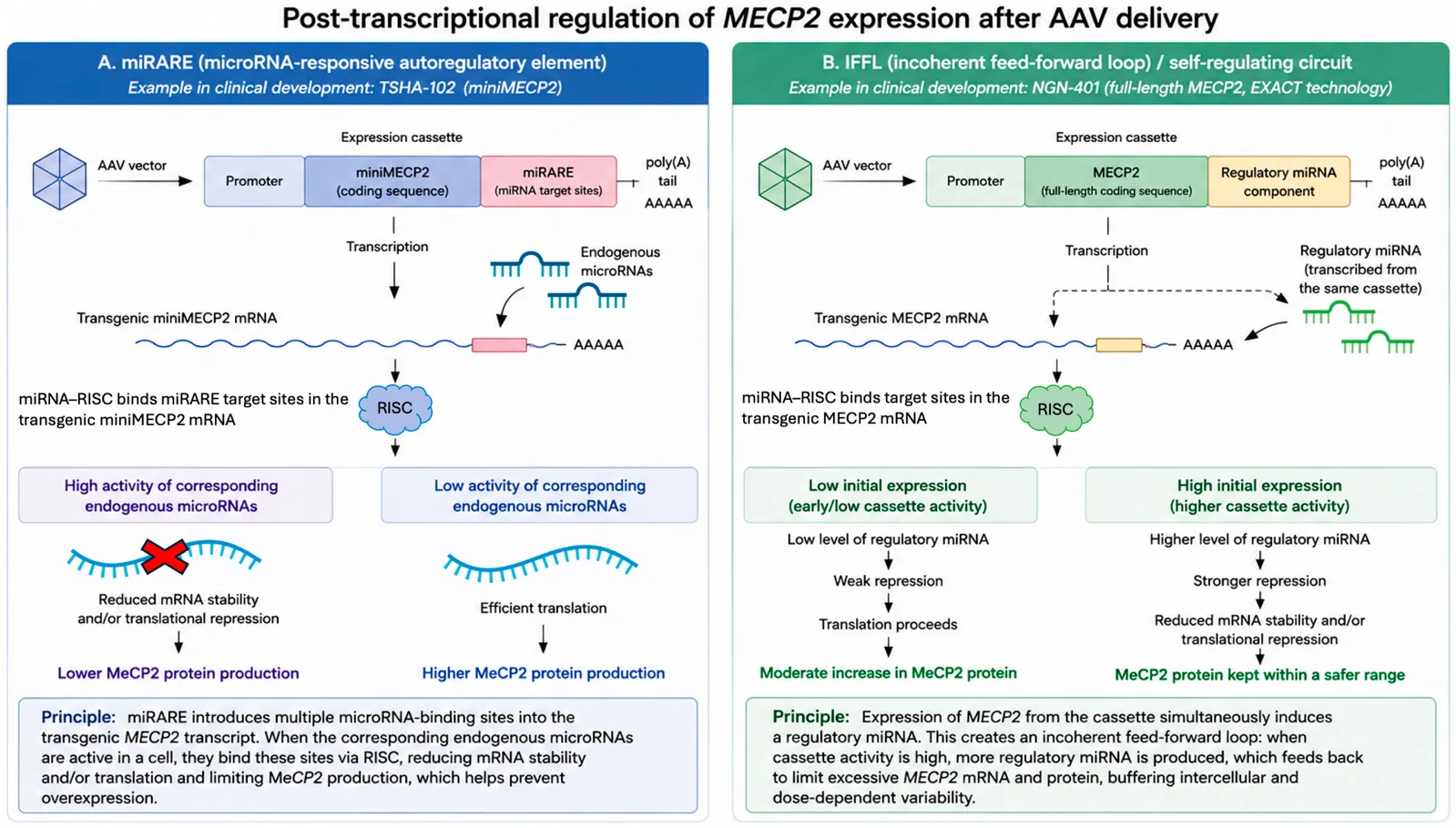
Conceptual scheme of miRNA-mediated post-transcriptional regulation of MeCP2 expression after AAV delivery. The figure illustrates two general regulatory principles designed to limit excessive MeCP2 production after transgene expression. (**A**) miRARE-based regulation. The transgenic MeCP2 or mini*MECP2* mRNA contains miRNA-responsive target sites. Endogenous miRNAs loaded into the RNA-induced silencing complex (RISC) can bind these sites and reduce mRNA stability and/or repress translation, thereby limiting MeCP2 production when the corresponding miRNA pathway is active. (**B**) IFFL/self-regulating miRNA-mediated circuit. In an incoherent feed-forward loop (IFFL), expression from the therapeutic cassette is coupled to a regulatory miRNA component that acts back on the transgenic *MECP2* mRNA through miRNA/RISC-mediated repression. This creates a buffering mechanism intended to reduce excessive or highly variable MeCP2 expression. RISC is shown as the common effector complex for miRNA-mediated post-transcriptional repression in both strategies. The scheme is conceptual and is not intended to represent all molecular details of specific proprietary clinical constructs. Neither approach should be interpreted as a direct physiological sensor of MeCP2 abundance or as full restoration of endogenous *MECP2*/*Mecp2* regulation.

In the published preclinical study this self-regulating construct improved phenotypic outcomes and reduced risks associated with variable transgene expression in a mouse model of RTT [44]. Clinically, this approach is represented by NGN-401, an AAV9-based investigational therapy designed to deliver full-length human *MECP2* using Neurogene’s EXACT transgene regulation technology. This distinction is important because NGN-401 does not use a truncated miniMECP2 product. NGN-401 is being evaluated in the EMBOLDEN study in girls with typical RTT [ClinicalTrials.gov: NCT05898620]. Publicly reported Phase 1/2 data from the sponsor have described developmental milestone gains and multidomain functional improvements in treated participants, including effects on hand function, gross motor function, and communication. These results appear promising, but they should be distinguished from peer-reviewed preclinical evidence and interpreted cautiously until full peer-reviewed clinical data and longer follow-up are available. The conceptual logic of miRARE-based and IFFL/self-regulating miRNA-mediated post-transcriptional control is summarized in Figure 2.

The comparison between TSHA-102 and NGN-401 is therefore not simply a comparison between two AAV9-based *MECP2* delivery platforms, but a comparison between two different solutions to the MeCP2 dosage problem. TSHA-102 prioritizes cassette compactness and post-transcriptional limitation of a miniMECP2 transcript through miRARE. This design may reduce the risk of excessive expression and allows inclusion of regulatory elements within the AAV cassette, but it depends on the assumption that miniMECP2 can provide sufficient functional correction over time. NGN-401 prioritizes preservation of the complete MeCP2 coding sequence and uses EXACT-based self-regulation to reduce the risk of overexpression. This design addresses the protein-completeness issue more directly, but its clinical interpretation depends on whether the regulatory cassette can maintain full-length MeCP2 within a safe range across heterogeneous CNS cell populations after intracerebroventricular delivery.

Thus, TSHA-102 and NGN-401 should not be considered interchangeable regulated AAV strategies. They differ in encoded MeCP2 product, route of administration, cassette architecture, and regulatory logic. For TSHA-102, key long-term questions include durability of miniMECP2-mediated benefit, stability of miRARE function, and adequacy of regulation across developmental stages and cell types. For NGN-401, key questions include whether full-length MeCP2 expression remains within a therapeutically acceptable range, whether EXACT-mediated regulation is sufficiently robust in vivo, and whether the benefits of full-length expression and direct CNS delivery outweigh procedure- and dose-related risks. Future comparison of these programs should therefore focus not only on early functional gains, but also on durability of clinical response, delayed toxicity, immune responses, CNS and peripheral biodistribution, and indirect evidence that MeCP2 expression remains neither insufficient nor excessive over time.

The main limitations of miRNA-responsive and miRNA-mediated systems are related to their dependence on cellular context. miRNA levels and RISC activity may differ across cell types, CNS regions, developmental stages, and disease states. Consequently, the same cassette may be regulated differently in distinct cell populations. This is particularly important for RTT, because the therapeutic target includes multiple neuronal subtypes and possibly selected glial populations, while also requiring consideration of cellular mosaicism in female patients. Therefore, evaluation of these systems should include not only the average MeCP2 level in tissue, but also the distribution of expression across cell types. An additional risk is the potential impact on endogenous miRNA pathways. Built-in miRNA-binding sites and synthetic miRNA circuits may compete with endogenous mRNAs for components of the miRNA/RISC machinery.

## 5. Peripheral MeCP2 Deficiency and Its Implications for Therapeutic Response

Although RTT is primarily considered a disorder of CNS function, MeCP2 is broadly expressed outside the nervous system. This is relevant for gene therapy because peripheral MeCP2 deficiency may contribute to the overall disease burden and may influence the apparent therapeutic response. Conditional mouse studies help define this relationship. Preservation of near-normal MeCP2 expression in the nervous system prevents many severe RTT-like neurological, behavioral, sensorimotor, gait, respiratory, and cardiac manifestations, supporting the CNS as the primary therapeutic target. However, severe depletion of *Mecp2* outside the nervous system still produces clinically relevant phenotypes, including hypoactivity, exercise fatigue, and bone abnormalities [45]. These findings suggest that CNS-directed correction may be sufficient to modify major neurological manifestations, but may not fully correct all peripheral or systemic components of the disorder.

Several peripheral systems may be relevant in this context. RTT patients frequently show non-neurological comorbidities, including gastrointestinal dysfunction, growth and nutritional abnormalities, reduced bone mineral density, orthopedic complications, metabolic alterations, cardiac involvement, and immune or inflammatory changes [46,47,48,49,50,51]. Some of these manifestations may be secondary to CNS dysfunction, impaired mobility, feeding difficulties, autonomic dysregulation, or medication exposure. However, experimental data also support more direct roles of MeCP2 in peripheral tissues. For example, liver-targeted *Mecp2* deletion in mice has been associated with fatty liver disease and dyslipidemia, implicating MeCP2 in the regulation of lipid metabolism outside the CNS [52]. MeCP2 has also been shown to regulate microglia and peripheral macrophage responses to inflammatory stimuli, indicating that immune and inflammatory components may contribute to the systemic phenotype [53].

This peripheral dimension has two important implications for therapeutic development. First, predominantly CNS-directed therapies may improve core neurological features while leaving some systemic manifestations incompletely corrected. Therefore, long-term clinical evaluation should include not only neurological and developmental endpoints, but also measures of growth, nutritional status, bone health, metabolic profile, autonomic and cardiac function, and inflammatory or immune-related biomarkers. Second, attempts to broaden vector distribution beyond the CNS must be balanced against the risk of peripheral overexpression and organ toxicity. In this sense, peripheral tissues represent both a potential therapeutic target and a potential source of safety concerns. For regulated *MECP2* gene therapy, the goal is therefore not simply to maximize CNS transduction, but to achieve a clinically meaningful balance between CNS efficacy, peripheral safety, and, where relevant, correction of systemic MeCP2-related dysfunction.

## 6. Experimental Inducible Systems

Inducible genetic systems, such as Cre-LoxP, CreERT2, and Tet-On/Tet-Off, are used primarily as experimental tools to study *Mecp2* function in model organisms. They allow *Mecp2* to be deleted or restored at a defined time point or in specific cell populations. However, data obtained with these systems should be distinguished from studies of therapeutic AAV construct delivery. In Cre-LoxP and CreERT2 models, the regulatory element is usually pre-engineered into the animal genome, whereas clinically oriented approaches must achieve delivery, expression, and transgene control after administration of a therapeutic vector. Therefore, inducible systems are important as proof-of-concept tools, but they are not, by themselves, clinically ready therapeutic platforms for RTT.

Cre-LoxP-dependent reactivation of *Mecp2* in an RTT mouse model showed in early studies that restoration of gene expression in animals can be associated with attenuation of several neurological abnormalities [11]. This finding refers to a specific experimental model in which a lox-Stop cassette had been pre-inserted into the endogenous *Mecp2* locus. Thus, it supports the possibility of phenotypic modification after *Mecp2* restoration in mice, but it does not address clinical delivery, dosage control, or MeCP2 safety in patients. Cre-LoxP approaches have also been used to assess the contribution of specific cell populations to RTT-like manifestations. Deletion of *Mecp2* in Sim1-expressing neurons was associated with changes in body weight, stress response, and aggressive behavior in mice, suggesting a contribution of this population to selected components of the phenotype [54]. Restoration of *Mecp2* in GABAergic neurons improved some behavioral and physiological measures in mice, supporting the importance of cellular context in the analysis of MeCP2 function [55].

Tet-On/Tet-Off systems represent a general approach to inducible transgene regulation using doxycycline-dependent regulatory elements [56]. However, published data demonstrating safe and dose-controllable use of Tet-On/Tet-Off systems for RTT therapy through in vivo regulation of *Mecp2* are currently lacking. Therefore, these systems should presently be considered a general experimental principle of inducible expression with limited direct evidence for this specific disorder. In this sense, inducible systems are useful for analyzing *Mecp2* function, the reversibility of selected phenotypic manifestations in mouse models, and the contribution of specific cell populations. However, their therapeutic relevance for RTT remains indirect: they help define requirements for future regulated platforms, but do not themselves solve the challenges of safe delivery, cellular selectivity, or maintaining MeCP2 within an acceptable expression range in patients.

## 7. XIST-Dependent X-Chromosome Inactivation

In female patients with RTT, the pathogenic variant usually affects one of the two copies of the X-linked *MECP2* gene. In female cells, one of the two X chromosomes undergoes inactivation during early embryonic development, a process known as X-chromosome inactivation (XCI). As a result, a transcriptionally repressed inactive X chromosome is formed and is referred to as Xi, whereas the active X chromosome is referred to as Xa. XCI is a dosage-compensation mechanism that limits the expression of most X-linked genes to one of the two X chromosomes in female mammals. However, some genes can escape inactivation and retain expression from Xi [57,58]. Because XCI occurs randomly with respect to the maternal or paternal X chromosome in most somatic cells, heterozygous female patients with RTT develop cellular mosaicism: some cells express the X chromosome carrying the functional *MECP2* copy, whereas others express the X chromosome carrying the pathogenic *MECP2* variant. If the functional *MECP2* copy is located on Xi, it remains transcriptionally silenced in that cell. Therefore, reactivation of endogenous *MECP2* from Xi is considered an alternative strategy for restoring MeCP2: instead of introducing an additional exogenous gene copy, the therapeutic intervention targets an existing but epigenetically silenced locus [59].

A key component of XCI is the long non-coding RNA XIST, or X-inactive specific transcript, which was originally considered a candidate gene associated with the X-inactivation center [57]. X-chromosome inactivation is maintained not only by XIST RNA, but also by several interconnected epigenetic mechanisms, including histone modifications, DNA methylation changes, and three-dimensional chromatin reorganization.

One experimental approach to Xi reactivation involves combined targeting of XIST RNA and DNA methylation. A combination of an antisense oligonucleotide against XIST RNA and a DNA methylation inhibitor increased *Mecp2* expression from Xi in cellular models, demonstrating the possibility of partial reactivation of Xi-linked *Mecp2* through simultaneous interference with RNA-dependent and epigenetic layers of silencing [17]. However, this result should not be interpreted as evidence of safe gene-specific reactivation of *MECP2*/*Mecp2*. Targeting XIST RNA and DNA methylation affects mechanisms involved in maintaining the broader Xi state and genomic silencing. Therefore, a major risk of this approach is potential reactivation not only of *MECP2*/*Mecp2*, but also of other X-linked genes, as well as possible effects of DNA methylation inhibition outside Xi.

Pharmacological screens have also shown that Xi-linked *Mecp2* can be sensitive to modulation of specific signaling and epigenetic regulators. shRNA library screening identified a link between BMP/TGF-β signaling, regulation of XIST expression, and reactivation of MeCP2 from Xi [60]. Pharmacological reactivation of Xi-linked *Mecp2* has also been described after targeting PI3K/AKT and BMP/TGF-β pathways in cellular models and in vivo in mice [61]. These data provide evidence that Xi-linked *Mecp2* is regulatable, but they also illustrate a general limitation of the approach: signaling inhibitors are not locus-specific tools for *MECP2*/*Mecp2* and may affect a broad range of cellular processes. Therefore, therapeutic interpretation of these findings requires evaluation not only of increased MeCP2 expression, but also of transcriptional consequences in other genes and tissues.

More locally targeted approaches to *MECP2*/*Mecp2* reactivation are discussed in the next section, because they are related not so much to general weakening of the XIST-dependent XCI program as to targeted epigenetic modulation of a specific locus. At the same time, one of the most important issues remains the selectivity of reactivation. Reactivation of Xi-linked genes depends on the local genetic and epigenetic context. Mira-Bontenbal et al. showed that reactivation of *Mecp2* and other X-linked genes depends on local genomic context, as it correlated with CpG density and distance to escapee genes [62]. CpG density reflects the enrichment of a genomic region in CpG dinucleotides, which are associated with regulatory regions and DNA methylation, whereas escapee genes are genes that partially retain expression from Xi [58,63]. Therefore, proximity to such genes may indicate a less stringently repressed chromatin environment [62]. In this context, if the same approach induces different levels of reactivation in different cells or affects neighboring X-linked genes, therapeutic evaluation cannot be limited to measuring MeCP2 alone. Data are needed on the transcriptomic profile of Xi, the extent of reactivation of other genes, and the stability of the effect in neuronal cells.

Several parameters are particularly important for future studies. First, it is necessary to quantify not only the average level of MeCP2, but also the distribution of expression between cells. Second, *MECP2*/*Mecp2* reactivation should be accompanied by analysis of other X-linked genes to determine the selectivity of the intervention. Third, results obtained in fibroblasts or reporter systems should be validated in neuronal models and, where possible, in vivo. Fourth, for epigenome editing approaches, off-target profiles should be evaluated not only at the sequence level, but also at the level of epigenetic state and transcriptome.

More locally targeted strategies for *MECP2*/*Mecp2* reactivation are based not on broad weakening of XIST-dependent silencing or systemic modulation of DNA methylation, but on delivery of epigenetic regulatory domains to selected regions of the locus. These strategies differ in their engineering logic and risk profile and are therefore discussed separately in the following section.

## 8. CRISPR/dCas9-Based Approaches

CRISPR-based approaches in the context of RTT can be broadly divided into two groups. The first involves DNA sequence editing aimed at correcting a pathogenic *MECP2*/*Mecp2* variant. The second does not alter the gene sequence itself, but instead regulates expression of the endogenous locus, for example through CRISPR activation, CRISPR interference, or epigenome editing using catalytically inactive Cas9, referred to as dCas9. The second group is particularly relevant to this review because it is linked to control of MeCP2 dosage and potential reactivation of endogenous *MECP2*/*Mecp2* without introducing an additional gene copy. In contrast to approaches targeting XIST RNA, global DNA methylation, or signaling pathways, CRISPR/dCas9-based systems can deliver regulatory domains to selected genomic regions. For example, dCas9-TET1 is an epigenome-editing system in which dCas9 does not cleave DNA but instead serves as a targeting module that recruits the TET1 demethylating domain to defined regulatory regions. In the context of RTT, this approach is of interest for local epigenetic reactivation of *MECP2*/*Mecp2* from Xi, although it does not eliminate concerns related to delivery, specificity, MeCP2 dosage control, and long-term safety. The general feasibility of using dCas9 as a platform for programmable transcriptional regulation was demonstrated in early studies, which provided an important methodological foundation for this field [64,65].

Direct sequence editing of *MECP2*/*Mecp2* could, in principle, correct the primary genetic defect; however, in RTT this approach faces several limitations. The mutational spectrum of *MECP2* in RTT includes different classes of pathogenic variants, including missense, nonsense, frameshift, and splice-site variants, as well as larger deletions [66]. Recent data from patient-derived human cortical organoids further support the functional relevance of mutation-specific effects: organoids carrying the R306C missense or V247X truncating *MECP2* variants showed abnormal neuronal activity and altered network organization, with the truncating V247X variant producing more severe disruption of functional responses and connectivity than R306C [67]. These findings reinforce the need to consider mutational heterogeneity when evaluating mutation-specific editing strategies. Despite the presence of recurrent mutations, this heterogeneity limits the applicability of strategies focused on correcting a single specific variant [68]. It also highlights the importance of local RTT patient registries that capture population-specific mutational patterns. In addition, for a neurological disorder, editing must occur in relevant CNS cell populations, and incomplete editing would result in mosaic restoration. Therefore, direct DNA editing should currently be considered a theoretical direction rather than a clinically ready strategy for MeCP2 dosage control in RTT.

A more relevant approach to the dosage problem is epigenome editing of endogenous *MECP2*/*Mecp2*. In contrast to nuclease-based editing, dCas9 systems can deliver regulatory domains to defined genomic regions without creating double-strand DNA breaks, which is a general principle of CRISPR-based epigenome editing. In the context of RTT, this is particularly important for reactivating the functional *MECP2*/*Mecp2* copy on the inactive X chromosome. In the study by Qian et al. (2023), this approach was applied to reactivate *MECP2* from the inactive X chromosome in human RTT hESCs and hESC-derived neurons [69]. The authors used multiplex epigenome editing, including targeted demethylation of the *MECP2* promoter with dCas9-TET1. Promoter demethylation led to reactivation of *MECP2* from Xi in RTT hESCs without detectable off-target transcriptional effects within the scope of the analysis performed. In neurons derived from edited hESCs, *MECP2* reactivation was maintained and was accompanied by partial correction of cellular abnormalities, including reduced soma size and electrophysiological disturbances described by the authors as RTT-associated neuronal defects. These data are important as a proof of concept for local epigenetic reactivation of *MECP2* in human cellular models of RTT, but they should not yet be interpreted as evidence of in vivo efficacy or safety. Key unresolved issues include delivery of dCas9-TET1 and guide RNAs to relevant CNS cells, persistence of the epigenetic effect over time, control of restored MeCP2 levels, and potential off-target effects beyond the models tested. In addition, the absence of detectable off-target transcriptional effects in this analysis does not exclude off-target risks under other delivery conditions, doses, or durations of epigenetic editor expression.

A separate issue is dosage control after reactivation of the endogenous locus. Reactivation of *MECP2*/*Mecp2* from Xi may be closer to the natural regulatory context than exogenous AAV-mediated delivery, because expression occurs from the native genomic locus. However, even in this case, MeCP2 levels may depend on the extent of demethylation, the number of affected cells, the local chromatin state, and the stability of the epigenetic modification. Therefore, epigenome editing should not automatically be considered a fully self-regulating system.

CRISPR/dCas9 and epigenome editing represent an important experimental direction for controlling endogenous *MECP2*/*Mecp2*, especially in the context of reactivating the Xi-linked allele. Their potential advantage lies in the attempt to restore expression from the native locus rather than introduce an additional gene copy. However, this direction remains largely experimental. Further evaluation will require in vivo data, quantitative analysis of MeCP2 distribution between cells, assessment of off-target epigenetic effects, evaluation of selectivity toward other X-linked genes, and development of safe delivery systems for the CNS.

**Figure 3:**
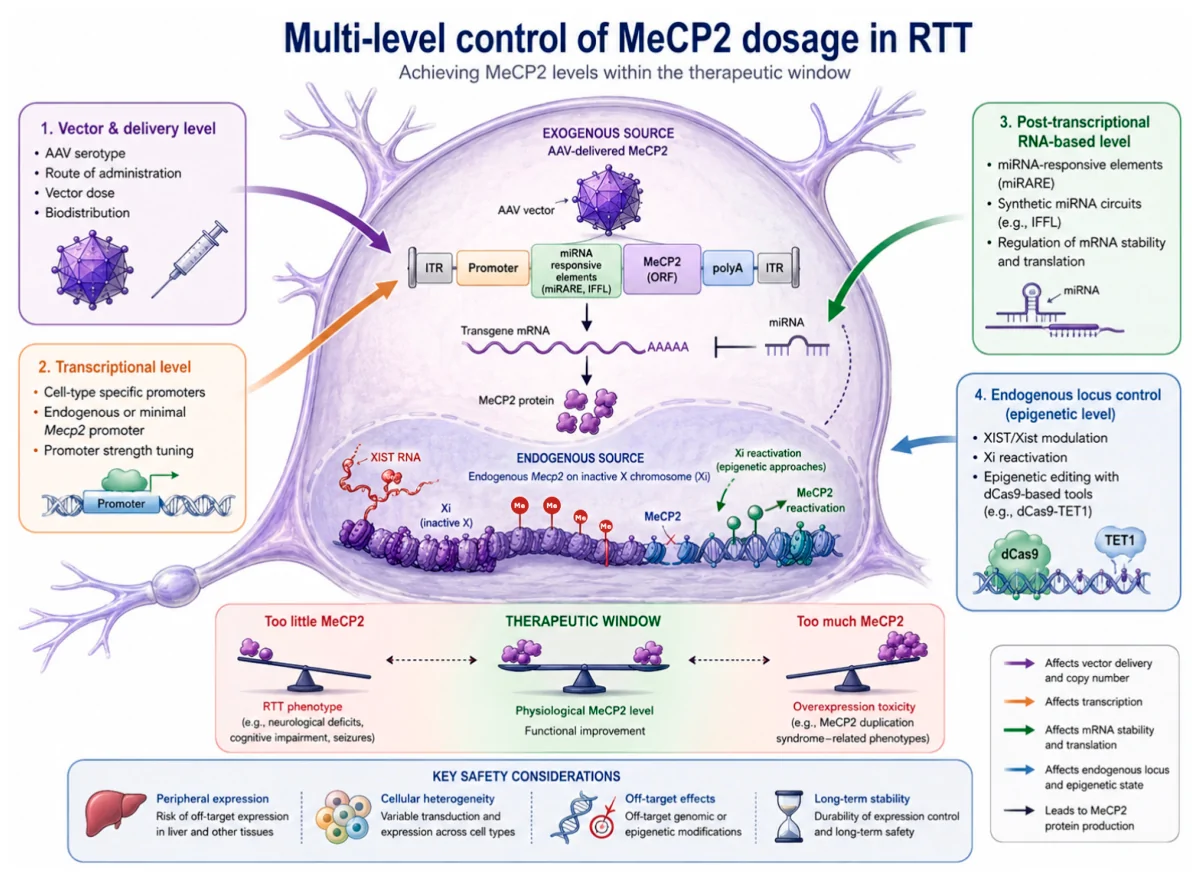
Multilayered control of MeCP2 dosage in RTT. Schematic representation of the main regulatory layers involved in controlling MeCP2 expression in the context of developing safer genetic strategies for RTT. The center of the figure shows a CNS cell with two potential sources of MeCP2: an exogenous AAV expression cassette and the endogenous *MECP2*/*Mecp2* locus on the inactive X chromosome (Xi). Expression can be controlled at several levels, including vector delivery parameters, promoter selection, post-transcriptional regulation mediated by miRNA-responsive elements, and reactivation of endogenous *MECP2*/*Mecp2* from Xi through XIST-dependent or dCas9-mediated epigenetic approaches. The lower part of the figure illustrates the therapeutic window for MeCP2: insufficient protein levels are associated with the RTT phenotype, whereas excessive expression may be associated with toxicity and phenotypes related to *MECP2*/*Mecp2* duplication. Key safety parameters that require evaluation during the development of such strategies are also shown, including peripheral expression, cellular heterogeneity, off-target effects, and long-term stability of regulation.

The approaches discussed above differ in the level at which they restrict MeCP2 expression: delivery determines the distribution of the therapeutic construct across tissues and cells, the promoter defines baseline transcriptional activity, miRNA-responsive elements act at the level of transgenic mRNA, and Xi reactivation and dCas9-based epigenome editing target the endogenous *MECP2*/*Mecp2* locus. These strategies are not interchangeable, because each can reduce one set of risks while introducing another. A general scheme of multilayered MeCP2 dosage control is presented in Figure 3, and a comparative summary of the main approaches is provided in Table 1.

## 9. Conclusions

MeCP2 dosage sensitivity remains a key limitation in the development of genetic strategies for RTT. Therefore, the therapeutic goal is not to maximize MeCP2 expression, but to achieve a controlled protein level that is sufficient for functional correction while remaining safe with respect to the risk of overexpression. Non-coding RNA-based and epigenetic mechanisms may play an important role in such control, because they enable regulation of MeCP2 not only at the level of transgene delivery, but also at post-transcriptional or endogenous chromatin levels. miRNA-responsive elements, synthetic miRNA-mediated circuits, XIST-related approaches, and dCas9-based epigenome editing expand the range of possible strategies, but they require careful assessment of cell specificity, off-target effects, long-term stability, and effects on endogenous RNA and epigenetic mechanisms.

Clinical translation of regulated AAV-based approaches for RTT has already begun and is accompanied by the use of regulatory mechanisms intended to accelerate the development of therapies for severe rare diseases. In particular, the TSHA-102 and NGN-401 programs have received special FDA designations that allow closer interaction with the regulator and may facilitate clinical development. However, these designations do not mean that the therapies have already demonstrated efficacy or long-term safety. Therefore, further evaluation of MeCP2-targeted approaches should rely primarily on peer-reviewed clinical data, long-term follow-up, quantitative analysis of MeCP2 levels in relevant cell types, and monitoring for signs of both insufficient and excessive expression.

## Figures and Tables

**Table 1:** Comparative characteristics of the main strategic principles for controlling MeCP2 expression in RTT.

Criterion	Standard AAV-Mediated *MECP2*/*Mecp2* Delivery	AAV with Regulatory Elements	CRISPR/dCas9 and Epigenetic Regulation	Reactivation of *MECP2*/*Mecp2* from Xi
**Main principle**	Delivery of an exogenous *MECP2*/*Mecp2* copy	Delivery of *MECP2*/*Mecp2* with additional elements that limit expression	Modulation of the sequence or epigenetic state of the endogenous locus	Increased expression of endogenous *MECP2*/*Mecp2* from the inactive X chromosome
**Control of MeCP2 level**	Limited by vector dose, promoter, and route of administration; no built-in self-regulation	Additional post-transcriptional control, for example miRARE or IFFL	Potentially local modulation of the endogenous locus; the degree of dosage control requires further evaluation	Potential restoration of expression from the endogenous locus; the level of reactivation may be variable
**Cellular specificity**	Depends on serotype, route of administration, and promoter	Depends on serotype, promoter, and activity of the miRNA/RISC system	Depends on the delivery system, gRNA, and cellular state	Relevant to mosaicism in female patients, but depends on Xi accessibility and cellular context
**Main advantages**	Relatively simple construct; preclinical data are available for *MECP2*/*Mecp2* delivery	Reduces the risk of overexpression in preclinical models; some platforms have advanced to clinical evaluation	Allows targeting of the endogenous locus without introducing an additional coding copy in some designs	Does not require addition of an exogenous *MECP2*/*Mecp2* copy; theoretically accounts for mosaicism in female patients
**Main limitations**	Narrow therapeutic window; risk of peripheral expression; heterogeneous transduction	Dependence on cellular context and miRNA pathways; long-term stability requires evaluation	Challenging CNS delivery; off-target genomic or epigenetic effects; limited in vivo evidence for RTT	Risk of reactivating other X-linked genes; incomplete or variable reactivation; limited clinical maturity
**Risk of MeCP2 overexpression**	Increased at high vector doses or with strong expression	May be reduced through post-transcriptional control	Depends on the degree and selectivity of activation	Depends on the selectivity and level of reactivation
**Reversibility/flexibility**	Limited after administration	Limited despite additional regulation	Depends on the platform and type of intervention	May be partial with epigenetic intervention, but the stability of the effect requires evaluation
**Development stage**	Preclinical studies; dose- and safety-related limitations have been identified	Preclinical studies and clinical evaluation of selected platforms	Predominantly experimental stage	Predominantly experimental stage

## Data Availability

No new data were created or analyzed in this study. Data sharing is not applicable to this article.

## Abbreviations

The following abbreviations are used in this manuscript:

AAV	adeno-associated virus
CNS	central nervous system
CRISPR	clustered regularly interspaced short palindromic repeats
dCas9	catalytically dead (inactive) Cas9
EPSC	excitatory postsynaptic current
EXACT	Expression Attenuation via Construct Tuning
FDA	Food and Drug Administration
IFFL	incoherent feed-forward loop
lncRNA	long non-coding RNA
IPSC	inhibitory postsynaptic current
*MECP2*	methyl-CpG-binding protein 2 gene, human gene symbol
MeCP2	methyl-CpG-binding protein 2, protein
*Mecp2*	methyl-CpG-binding protein 2 gene, mouse gene symbol
miRNA	microRNA
miRARE	microRNA-responsive autoregulatory element
RISC	RNA-induced silencing complex
RTT	Rett syndrome
UTR	untranslated region
vg	vector genomes
XCI	X-chromosome inactivation
Xi	inactive X chromosome
XIST	X-inactive specific transcript

## References

[1] Neul J.L., Kaufmann W.E., Glaze D.G., Christodoulou J., Clarke A.J., Bahi-Buisson N., Leonard H., Bailey M.E.S., Schanen N.C., Zappella M. (2010). Rett Syndrome: Revised Diagnostic Criteria and Nomenclature. Ann. Neurol..

[2] Amir R.E., Van Den Veyver I.B., Wan M., Tran C.Q., Francke U., Zoghbi H.Y. (1999). Rett Syndrome Is Caused by Mutations in X-Linked MECP2, Encoding Methyl-CpG-Binding Protein 2. Nat. Genet..

[3] Chahrour M., Zoghbi H.Y. (2007). The Story of Rett Syndrome: From Clinic to Neurobiology. Neuron.

[4] Lyst M.J., Bird A. (2015). Rett Syndrome: A Complex Disorder with Simple Roots. Nat. Rev. Genet..

[5] Liyanage V.R.B., Rastegar M. (2014). Rett Syndrome and MeCP2. Neuromolecular Med..

[6] Frullanti E., Papa F.T., Grillo E., Clarke A., Ben-Zeev B., Pineda M., Bahi-Buisson N., Bienvenu T., Armstrong J., Roche Martinez A. (2019). Analysis of the Phenotypes in the Rett Networked Database. BMC Genom..

[7] Nan X., Bird A. (2001). The Biological Functions of the Methyl-CpG-Binding Protein MeCP2 and Its Implication in Rett Syndrome. Brain Dev..

[8] Mansooralavi N., Lowry W.E. (2026). New Insights into Rett Syndrome Pathogenesis: Defining the Role of MEPC2 in DNA Damage. Front. Neurol..

[9] Palmieri M., Pozzer D., Landsberger N. (2023). Advanced Genetic Therapies for the Treatment of Rett Syndrome: State of the Art and Future Perspectives. Front. Neurosci..

[10] Na E.S., Nelson E.D., Kavalali E.T., Monteggia L.M. (2012). The Impact of MeCP2 Loss- or Gain-of-Function on Synaptic Plasticity. Neuropsychopharmacology.

[11] Guy J., Gan J., Selfridge J., Cobb S., Bird A. (2007). Reversal of Neurological Defects in a Mouse Model of Rett Syndrome. Science.

[12] Collins A.L., Levenson J.M., Vilaythong A.P., Richman R., Armstrong D.L., Noebels J.L., Sweatt J.D., Zoghbi H.Y. (2004). Mild Overexpression of MeCP2 Causes a Progressive Neurological Disorder in Mice. Hum. Mol. Genet..

[13] Allison K., Maletic-Savatic M., Pehlivan D. (2024). MECP2-related disorders while gene-based therapies are on the horizon. Front. Genet..

[14] Good K.V., Vincent J.B., Ausió J. (2021). MeCP2: The Genetic Driver of Rett Syndrome Epigenetics. Front. Genet..

[15] Sharifi O., Yasui D.H. (2021). The Molecular Functions of MeCP2 in Rett Syndrome Pathology. Front. Genet..

[16] Sinnett S.E., Boyle E., Lyons C., Gray S.J. (2021). Engineered MicroRNA-Based Regulatory Element Permits Safe High-Dose MiniMECP2 Gene Therapy in Rett Mice. Brain.

[17] Carrette L.L.G., Wang C.Y., Wei C., Press W., Ma W., Kelleher R.J., Lee J.T. (2018). A Mixed Modality Approach towards Xi Reactivation for Rett Syndrome and Other X-Linked Disorders. Proc. Natl. Acad. Sci. USA.

[18] Van Esch H., Bauters M., Ignatius J., Jansen M., Raynaud M., Hollanders K., Lugtenberg D., Bienvenu T., Jensen L.R., Gécz J. (2005). Duplication of the MECP2 Region Is a Frequent Cause of Severe Mental Retardation and Progressive Neurological Symptoms in Males. Am. J. Hum. Genet..

[19] Na E.S., Nelson E.D., Adachi M., Autry A.E., Mahgoub M.A., Kavalali E.T., Monteggia L.M. (2012). A Mouse Model for MeCP2 Duplication Syndrome: MeCP2 Overexpression Impairs Learning and Memory and Synaptic Transmission. J. Neurosci..

[20] Collins B.E., Neul J.L. (2022). Rett Syndrome and MECP2 Duplication Syndrome: Disorders of MeCP2 Dosage. Neuropsychiatr. Dis. Treat..

[21] D’Mello S.R. (2021). MECP2 and the Biology of MECP2 Duplication Syndrome. J. Neurochem..

[22] Bodda C., Tantra M., Mollajew R., Arunachalam J.P., Laccone F.A., Can K., Rosenberger A., Mironov S.L., Ehrenreich H., Mannan A.U. (2013). Mild Overexpression of Mecp2 in Mice Causes a Higher Susceptibility toward Seizures. Am. J. Pathol..

[23] Luoni M., Kubacki M., Giannelli S.G., Morosi F., Di Berardino C., Iannielli A., Sessa A., Colasante G., Di Patrizio Soldateschi E., Lanzuolo C. (2026). MeCP2 Gene Dosage-Dependent Neurodevelopmentally Restricted Defects Arise by Aberrant Activation of Cell Fate-Determining Bivalent Genes. Nat. Commun..

[24] Hoffbuhr K.C., Moses L.M., Jerdonek M.A., Naidu S., Hoffman E.P. (2002). Associations between MeCP2 Mutations, X-Chromosome Inactivation, and Phenotype. Ment. Retard. Dev. Disabil. Res. Rev..

[25] Braunschweig D., Simcox T., Samaco R.C., LaSalle J.M. (2004). X-Chromosome Inactivation Ratios Affect Wild-Type MeCP2 Expression within Mosaic Rett Syndrome and Mecp2−/+ Mouse Brain. Hum. Mol. Genet..

[26] Young J.I., Zoghbi H.Y. (2004). X-Chromosome Inactivation Patterns Are Unbalanced and Affect the Phenotypic Outcome in a Mouse Model of Rett Syndrome. Am. J. Hum. Genet..

[27] Bassuk A.G. (2021). Gene Therapy for Rett Syndrome. Genes Brain Behav..

[28] Bulcha J.T., Wang Y., Ma H., Tai P.W.L., Gao G. (2021). Viral Vector Platforms within the Gene Therapy Landscape. Signal Transduct. Target. Ther..

[29] Gadalla K.K., Bailey M.E., Spike R.C., Ross P.D., Woodard K.T., Kalburgi S.N., Bachaboina L., Deng J.V., West A.E., Samulski R.J. (2013). Improved Survival and Reduced Phenotypic Severity Following AAV9/MECP2 Gene Transfer to Neonatal and Juvenile Male Mecp2 Knockout Mice. Mol. Ther..

[30] Sinnett S.E., Hector R.D., Gadalla K.K.E., Heindel C., Chen D., Zaric V., Bailey M.E.S., Cobb S.R., Gray S.J. (2017). Improved MECP2 Gene Therapy Extends the Survival of MeCP2-Null Mice without Apparent Toxicity after Intracisternal Delivery. Mol. Ther. Methods Clin. Dev..

[31] Gadalla K.K.E., Vudhironarit T., Hector R.D., Sinnett S., Bahey N.G., Bailey M.E.S., Gray S.J., Cobb S.R. (2017). Development of a Novel AAV Gene Therapy Cassette with Improved Safety Features and Efficacy in a Mouse Model of Rett Syndrome. Mol. Ther. Methods Clin. Dev..

[32] Matagne V., Borloz E., Ehinger Y., Saidi L., Villard L., Roux J.C. (2021). Severe Offtarget Effects Following Intravenous Delivery of AAV9-MECP2 in a Female Mouse Model of Rett Syndrome. Neurobiol. Dis..

[33] Ageeva T., Shigapova R., Davletshin E., Plotnikova E., Rizvanov A., Mukhamedshina Y., Ageeva T., Shigapova R., Davletshin E., Plotnikova E. (2026). AAV9-Mediated Targeting of Defined Neuronal Populations in Spinal Cord Through Intrathecal Injection. Front. Biosci.-Elite.

[34] Kawashima T., Okuno H., Bito H. (2014). A New Era for Functional Labeling of Neurons: Activity-Dependent Promoters Have Come of Age. Front. Neural Circuits.

[35] Gallo F.T., Katche C., Morici J.F., Medina J.H., Weisstaub N.V. (2018). Immediate Early Genes, Memory and Psychiatric Disorders: Focus on c-Fos, Egr1 and Arc. Front. Behav. Neurosci..

[36] Sinnett S.E., Gray S.J. (2017). Recent Endeavors in MECP2 Gene Transfer for Gene Therapy of Rett Syndrome. Discov. Med..

[37] Jagadeeswaran I., Oh J., Sinnett S.E. (2024). Preclinical Milestones in MECP2 Gene Transfer for Treating Rett Syndrome. Dev. Neurosci..

[38] Dhungel B., Ramlogan-Steel C.A., Steel J.C. (2018). MicroRNA-Regulated Gene Delivery Systems for Research and Therapeutic Purposes. Molecules.

[39] Geisler A., Fechner H. (2016). MicroRNA-Regulated Viral Vectors for Gene Therapy. World J. Exp. Med..

[40] Bartel D.P. (2009). MicroRNAs: Target Recognition and Regulatory Functions. Cell.

[41] Sadhu C., Lyons C., Oh J., Jagadeeswaran I., Gray S.J., Sinnett S.E. (2024). The Efficacy of a Human-Ready MiniMECP2 Gene Therapy in a Pre-Clinical Model of Rett Syndrome. Genes.

[42] Osella M., Bosia C., Corá D., Caselle M. (2011). The Role of Incoherent MicroRNA-Mediated Feedforward Loops in Noise Buffering. PLoS Comput. Biol..

[43] Flynn M.J., Mayfield A.M.H., Du R., Gradinaru V., Elowitz M.B. (2024). Synthetic Dosage-Compensating MiRNA Circuits Allow Precision Gene Therapy for Rett Syndrome. bioRxiv.

[44] Ross P.D., Gadalla K.K.E., Thomson S.R., Selfridge J., Bahey N.G., Benito J., Burstein S.R., McMinn R., Bolon B., Hector R.D. (2025). Self-Regulating Gene Therapy Ameliorates Phenotypes and Overcomes Gene Dosage Sensitivity in a Mouse Model of Rett Syndrome. Sci. Transl. Med..

[45] Ross P.D., Guy J., Selfridge J., Kamal B., Bahey N., Elizabeth Tanner K., Gillingwater T.H., Jones R.A., Loughrey C.M., McCarroll C.S. (2016). Exclusive Expression of MeCP2 in the Nervous System Distinguishes between Brain and Peripheral Rett Syndrome-like Phenotypes. Hum. Mol. Genet..

[46] Vashi N., Justice M.J. (2019). Treating Rett Syndrome: From Mouse Models to Human Therapies. Mamm. Genome.

[47] Hara M., Takahashi T., Mitsumasu C., Igata S., Takano M., Minami T., Yasukawa H., Okayama S., Nakamura K., Okabe Y. (2015). Disturbance of Cardiac Gene Expression and Cardiomyocyte Structure Predisposes Mecp2-Null Mice to Arrhythmias. Sci. Rep..

[48] Ward C.S., Huang T.W., Herrera J.A., Samaco R.C., Pitcher M.R., Herron A., Skinner S.A., Kaufmann W.E., Glaze D.G., Percy A.K. (2016). Loss of MeCP2 Causes Urological Dysfunction and Contributes to Death by Kidney Failure in Mouse Models of Rett Syndrome. PLoS ONE.

[49] Justice M.J., Buchovecky C.M., Kyle S.M., Djukic A. (2013). A Role for Metabolism in Rett Syndrome Pathogenesis. Rare Dis..

[50] Pepe G., Coco R., Corica D., Luppino G., Morabito L.A., Lugarà C., Abbate T., Zirilli G., Aversa T., Stagi S. (2024). Endocrine Disorders in Rett Syndrome: A Systematic Review of the Literature. Front. Endocrinol..

[51] Pepe G., Coco R., Corica D., Di Rosa G., Bossowski F., Skorupska M., Aversa T., Stagi S., Wasniewska M. (2024). Prevalence of Endocrinopathies in a Cohort of Patients with Rett Syndrome: A Two-Center Observational Study. Genes.

[52] Kyle S.M., Saha P.K., Brown H.M., Chan L.C., Justice M.J. (2016). MeCP2 Co-Ordinates Liver Lipid Metabolism with the NCoR1/HDAC3 Corepressor Complex. Hum. Mol. Genet..

[53] Cronk J.C., Derecki N.C., Ji E., Xu Y., Lampano A.E., Smirnov I., Baker W., Norris G.T., Marin I., Coddington N. (2015). Methyl-CpG Binding Protein 2 Regulates Microglia and Macrophage Gene Expression in Response to Inflammatory Stimuli. Immunity.

[54] Fyffe S.L., Neul J.L., Samaco R.C., Chao H.T., Ben-Shachar S., Moretti P., McGill B.E., Goulding E.H., Sullivan E., Tecott L.H. (2008). Deletion of Mecp2 in Sim1-Expressing Neurons Reveals a Critical Role for MeCP2 in Feeding Behavior, Aggression, and the Response to Stress. Neuron.

[55] Ure K., Lu H., Wang W., Ito-Ishida A., Wu Z., He L.J., Sztainberg Y., Chen W., Tang J., Zoghbi H.Y. (2016). Restoration of Mecp2 Expression in GABAergic Neurons Is Sufficient to Rescue Multiple Disease Features in a Mouse Model of Rett Syndrome. Elife.

[56] Das A.T., Tenenbaum L., Berkhout B. (2016). Tet-On Systems For Doxycycline-Inducible Gene Expression. Curr. Gene Ther..

[57] Brown C.J., Ballabio A., Rupert J.L., Lafreniere R.G., Grompe M., Tonlorenzi R., Willard H.F. (1991). A Gene from the Region of the Human X Inactivation Centre Is Expressed Exclusively from the Inactive X Chromosome. Nature.

[58] Fang H., Disteche C.M., Berletch J.B. (2019). X Inactivation and Escape: Epigenetic and Structural Features. Front. Cell Dev. Biol..

[59] Grimm N.B., Lee J.T. (2022). Selective Xi Reactivation and Alternative Methods to Restore MECP2 Function in Rett Syndrome. Trends Genet..

[60] Sripathy S., Leko V., Adrianse R.L., Loe T., Foss E.J., Dalrymple E., Lao U., Gatbonton-Schwager T., Carter K.T., Payer B. (2017). Screen for Reactivation of MeCP2 on the Inactive X Chromosome Identifies the BMP/TGF-β Superfamily as a Regulator of XIST Expression. Proc. Natl. Acad. Sci. USA.

[61] Przanowski P., Wasko U., Zheng Z., Yu J., Sherman R., Zhu L.J., McConnell M.J., Tushir-Singh J., Green M.R., Bhatnagar S. (2018). Pharmacological Reactivation of Inactive X-Linked Mecp2 in Cerebral Cortical Neurons of Living Mice. Proc. Natl. Acad. Sci. USA.

[62] Mira-Bontenbal H., Tan B., Gontan C., Goossens S., Boers R.G., Boers J.B., Dupont C., van Royen M.E., IJcken W.F.J., French P. (2022). Genetic and Epigenetic Determinants of Reactivation of Mecp2 and the Inactive X Chromosome in Neural Stem Cells. Stem Cell Rep..

[63] Posynick B.J., Brown C.J. (2019). Escape from X-Chromosome Inactivation: An Evolutionary Perspective. Front. Cell Dev. Biol..

[64] Gilbert L.A., Larson M.H., Morsut L., Liu Z., Brar G.A., Torres S.E., Stern-Ginossar N., Brandman O., Whitehead E.H., Doudna J.A. (2013). CRISPR-Mediated Modular RNA-Guided Regulation of Transcription in Eukaryotes. Cell.

[65] Gilbert L.A., Horlbeck M.A., Adamson B., Villalta J.E., Chen Y., Whitehead E.H., Guimaraes C., Panning B., Ploegh H.L., Bassik M.C. (2014). Genome-Scale CRISPR-Mediated Control of Gene Repression and Activation. Cell.

[66] Wan M., Lee S.S.J., Zhang X., Houwink-Manville I., Song H.R., Amir R.E., Budden S., Naidu S.B., Pereira J.L.P., Lo I.F.M. (1999). Rett Syndrome and beyond: Recurrent Spontaneous and Familial MECP2 Mutations at CpG Hotspots. Am. J. Hum. Genet..

[67] Osaki T., Delepine C., Osako Y., Kranz D., Levin A., Nelson C., Fagiolini M., Sur M. (2026). Early Differential Impact of MeCP2 Mutations on Functional Networks in Rett Syndrome Patient-Derived Human Cortical Organoids. Nat. Commun..

[68] Gauthier J., De Amorim G., Mnatzakanian G.N., Saunders C., Vincent J.B., Toupin S., Kauffman D., St.-Onge J., Laurent S., Macleod P.M. (2005). Clinical Stringency Greatly Improves Mutation Detection in Rett Syndrome. Can. J. Neurol. Sci..

[69] Qian J., Guan X., Xie B., Xu C., Niu J., Tang X., Li C.H., Colecraft H.M., Jaenisch R., Liu X.S. (2023). Multiplex Epigenome Editing of MECP2 to Rescue Rett Syndrome Neurons. Sci. Transl. Med..

